# Role of BK_Ca_ in Stretch-Induced Relaxation of Colonic Smooth Muscle

**DOI:** 10.1155/2016/9497041

**Published:** 2016-11-28

**Authors:** Jie Ren, Fang Xin, Ping Liu, Hai-Yan Zhao, Si-Tao Zhang, Peng Han, Hai-Xia Huang, Wei Wang

**Affiliations:** ^1^Department of Physiology and Pathophysiology, School of Basic Medical Sciences, Capital Medical University, Beijing 100069, China; ^2^Yanjing Medical College, Capital Medical University, Beijing 101300, China

## Abstract

Stretch-induced relaxation has not been clearly identified in gastrointestinal tract. The present study is to explore the role of large conductance calcium-activated potassium channels (BK_Ca_) in stretch-induced relaxation of colon. The expression and currents of BK_Ca_ were detected and the basal muscle tone and contraction amplitude of colonic smooth muscle strips were measured. The expression of BK_Ca_ in colon is higher than other GI segments (*P* < 0.05). The density of BK_Ca_ currents was very high in colonic smooth muscle cells (SMCs). BK_Ca_ in rat colonic SMCs were sensitive to stretch. The relaxation response of colonic SM strips to stretch was attenuated by charybdotoxin (ChTX), a nonspecific BK_Ca_ blocker (*P* < 0.05). After blocking enteric nervous activities by tetrodotoxin (TTX), the stretch-induced relaxation did not change (*P* > 0.05). Still, ChTX and iberiotoxin (IbTX, a specific BK_Ca_ blocker) attenuated the relaxation of the colonic muscle strips enduring stretch (*P* < 0.05). These results suggest stretch-activation of BK_Ca_ in SMCs was involved in the stretch-induced relaxation of colon. Our study highlights the role of mechanosensitive ion channels in SMCs in colon motility regulation and their physiological and pathophysiological significance is worth further study.

## 1. Introduction

Mechanical stretch is a basic physiological stimulation. In addition to storage, digestion, absorption, and transport functions, the gastrointestinal (GI) tract is also a stress-sensing system and is often subjected to mechanical stretch stimulation [1, 2]. Different segments of the GI tract have different responses to stress stimulation: the chyme in the small bowel can lead to intestinal smooth muscle cells (SMCs) contraction by stretch-activation of calcium channels [3] that is conducive to digestion and absorption, while food in the stomach and food residue in the colon can induce a relaxation response, and the dilation of the smooth muscle ensures that increased contents in these cavities do not significantly increase chamber pressure, thus delaying emptying and, consequently, playing a role in storage [4]. The underlying mechanism is unclarified.

Different from the myogenic response of vessels, which refers to the contraction of arterioles initiated by elevations of transmural pressure without the involvement of neural and humoral factors [5], many studies have shown that direct stretch can cause the relaxation of lower esophageal sphincter [6, 7], colorectum [8, 9], and colon [10]. The relative mechanism of myogenic response of vessels is that the smooth muscle of the blood vessels reacts to the stretch by opening ion channels, which cause depolarization, leading to muscle contraction [5]. It has been reported that the relaxation of GI tract induced by stretch is based on nervous regulation and the localized distension activates enteric reflex to evoke relaxation [8, 10–12]. In spite of the neurogenic regulation, it is also reported that there are stretch-activated potassium ion channels in SMCs of GI tract [4]. However, the role of these channels in stretch-induced relaxation of GI tract at tissue level remains uncertain.

The myogenic response of arterioles involves stretch-activation of mechanosensitive ion channels resulting in depolarization of vascular SMCs and calcium influx through L-type voltage-gated calcium channels (VGCC) [13]. Some stretch-dependent potassium (SDK) currents have been found in colonic SMCs [4, 10, 14]. The activation of SDK outward currents could decrease excitability of SMCs and induce relaxation. Yet the molecular basis is poorly understood.

Large conductance calcium-activated potassium channels (BK_Ca_) are widely distributed in various tissues, including the smooth muscle of mammals. They are activated by depolarization and intracellular calcium with high potassium selectivity and high conductance. Potassium outflow mediated by even a mild activation of BK_Ca_ leads to obvious hyperpolarization and relaxation of smooth muscle. Recent studies reported that BK_Ca_ could be activated by membrane stretch [15–17]. Our previous study also found that BK_Ca_ in mouse colonic SMCs could be activated by stretch too [18]. Although the physiological significance of the mechanical gating of BK_Ca_ is unclear, it is reasonable to presume that BK_Ca_ in colonic SMCs would be activated when the colon wall is expanded as colonic content increases and result relaxation.

The present study investigated the expression and mechanosensitivity of BK_Ca_ in rat colonic SMCs and examined whether BK_Ca_ are involved in the stretch-induced relaxation of colonic smooth muscle.

## 2. Methods and Materials

### 2.1. Ethical Approval

All animal experimental procedures were approved by the Institutional Animal Care and Use Committee of the Capital Medical University, Beijing, China, and were performed in accordance with the “Regulations for the Administration of Affairs Concerning Experimental Animals (the State Science and Technology Commission, China, 1988).”

### 2.2. Protein Extraction and Western Blot Analysis

Eight-week-old male adult Sprague-Dawley (SD) rats (220 ± 10 g) were euthanized by CO_2_ according to an IACUC-approved protocol. The tissues was isolated and placed in a dish with ice-cold Krebs-Ringer solution containing (in mM) 117 NaCl, 24.8 NaHCO_3_, 4.7 KCl, 1.2 MgCl_2_, 1.2 KH_2_PO_4_, 2.56 CaCl_2_·2H_2_O, and 11.1 glucose (pH 7.35–7.40), incised along the mesenteric border, and pinned in a dish with the mucosa facing up. After removing the mucosa and submucosa by sharp dissection, total proteins were extracted from the stomach, duodenum, ileum, and colon smooth muscle layers with lysis buffer containing (in mM) 50 Tris·HCl (pH 7.5), 50 NaF, 2 EDTA, 2 EGTA, 0.1 sodium orthovanadate, and 1 DTT with 2% SDS and 15% protease inhibitor cocktail (Roche Germany) containing 1 mM phenylmethylsulfonyl fluoride (Roche, Mannheim, Germany). Equal volumes of protein lysates were separated by 10% SDS-PAGE and electrotransferred onto a nitrocellulose membrane (Millipore Ireland, County Cork, Ireland). The membrane was incubated overnight at 4°C with polyclonal antibodies against the *α*-subunit of BK_Ca_ (1 : 200; APC-021, Alomone, Jerusalem, Israel) and glyceraldehyde-3-phosphate dehydrogenase (GAPDH) (1 : 5000; G9545, Sigma-Aldrich, St. Louis, MO, USA) diluted in 5% nonfat dried milk. After three washes for 5 minutes with Tris-buffered saline (TBS), the membrane was incubated with IRDye TM700 (red)-conjugated affinity-purified anti-rabbit IgG (1 : 5000; 31004, Rockland, Philadelphia, PA, USA) for 1 hour at room temperature and then washed three times with TBS-0.05% Tween 20 (TBST) and twice with TBS. Detection was performed using the Odyssey infrared imaging system (LI-COR Biotechnology, Lincoln, NE, USA). The experiment was repeated six times.

### 2.3. Patch-Clamp Experiments

The SD rats were euthanized by CO_2_ according to an IACUC-approved protocol. The distal colon was quickly removed, washed with Ca^2+^-free Hank's solution, and pinned out in a Sylgard-lined dish. The Ca^2+^-free Hank's solution contained (in mM) 125 NaCl, 5.36 KCl, 15.5 NaHCO_3_, 0.336 Na_2_HPO_4_, 0.44 KH_2_PO_4_, 10 glucose, 2.9 sucrose, and 11 HEPES and was buffered to pH 7.4 with NaOH. After removal of the mucosa and submucosa, strips of smooth muscle layers were cut into small pieces and incubated in Ca^2+^-free Hank's solution containing 4 mg/ml fatty acid-free bovine serum albumin (Fraction V; Sigma-Aldrich, St. Louis, MO, USA), 14 U/ml papain from Carica papaya (Sigma-Aldrich, St. Louis, MO, USA), 230 U/ml collagenase (type II; Worthington, Freehold, NJ, USA), and 1 mM trypsin inhibitor (Sigma-Aldrich, St. Louis, MO, USA). The muscle fragments were digested in the enzyme solution at 37°C for 15 minutes and washed with Ca^2+^-free Hank's solution to create a cell suspension. All solutions were equilibrated with a mixture of 95% O_2_ and 5% CO_2_.

Patch-clamp studies were performed at room temperature, and cells were used within six hours after enzymatic isolation. The patch pipettes were made from borosilicate glass (BF150-110-10; Sutter Instruments, Novato, CA, USA). The electrode resistance was 8–10 MΩ for single-channel recording.

The single-channel activity was recorded in cell-attached configurations. The pipette solution and bath solution were the same, containing (in mM) 140 potassium aspartate, 5 EGTA, 2 MgCl_2_, and 10 HEPES, with pH adjusted to 7.4. The channel currents were amplified, filtered at 10 kHz, and digitized at 20 kHz using a data acquisition system (Axopatch-200A, Digidata1322A and pClamp 9.2; Axon Instruments, Union City, CA, USA). Suction steps for cell-attached patches were delivered to a side port of the pipette holder with a pressure-generating device (DALE20 pneumatic transducer tester; Luke Biomedical, Liverpool, UK) at a resolution of 1 mmHg. The pressure increased by 10 mmHg in turn and could be manually set in about 1 second. Single-channel activities were analyzed with the QUB software at 2 kHz (http://www.qub.buffalo.edu/; Buffalo, NY, USA). For each recording, 8-second data were carefully analyzed to obtain the channel current amplitude and the open probability. The channel activity in one patch was expressed as *NP*_*O*_, where *N* represents the number of channels in the patch and *P*_*o*_ represents the open probability of a single channel. The outward current was routinely shown to be upward.

### 2.4. Tension Recording of Colonic Smooth Muscle Strips

The distal colon was isolated from the SD rat and placed in a dish with ice-cold Krebs-Ringer which was gassed with 95% O_2_ and 5% CO_2_. The colon has an inner circular and outer longitudinal smooth muscle layers. The longitudinal muscle (LM) strips (2 × 8 mm; width × length) or circular muscle (CM) strips were cut along the direction of the longitudinal or circular axis. The fresh smooth muscle strips were prepared and mounted vertically in Krebs-Ringer's solution maintained at 37°C. One end of the strip was tied to a hook on the bottom of the organ bath and the other end was connected to a force transducer (MLT0201, Panlab company, Barcelona, Spain). The tissue strips were stretched to 1,000 mg of initial tension and equilibrated for 60 min before experiments were initiated. Parameters of the contraction amplitude and muscle tone of the colon strips were analyzed using the LabChart7 system (AD Instruments Pty Ltd., Bella Vista, New South Wales, Australia). Charybdotoxin (ChTX, 100 nM, Sigma-Aldrich, St. Louis, MO, USA), a nonspecific BK_Ca_ blocker, iberiotoxin (IbTX, 100 nM, Sigma-Aldrich, St. Louis, MO, USA), a specific BK_Ca_ blocker, and tetraethylammonium (TEA, 10 mM, Sigma-Aldrich, St. Louis, MO, USA), a broad-spectrum voltage-gated potassium channel (Kv) blocker, were used to block BK_Ca_. Voltage-gated sodium channel blocker tetrodotoxin (TTX, 100 nM, MedChemExpress, Monmouth Junction, NJ, USA) was used to eliminate the influence of the enteric nervous system.

For the stretch experiments, after pretreatment period, the strips were elongated 1 mm for 10 minutes each time and were stretched five times and the muscle tone was detected to study the response to stretch. The relative change of the muscle tone was determined by measuring the phasic contraction baseline curve.

### 2.5. Statistical Analysis

All data are expressed as means ± SEM. The data were analysed with GraphPad Prism 5.01 software (GraphPad Software, La Jolla, CA). The Student's paired* t*-test was used to compare patch-clamp data before and after intervention. For other data, one way ANOVA followed by Newman-Keuls test was used. For all analyses, statistical significance was accepted when* P* < 0.05.

## 3. Results

### 3.1. Expression of BK_Ca_ in Rat Colonic Smooth Muscle

**Figure 1:**
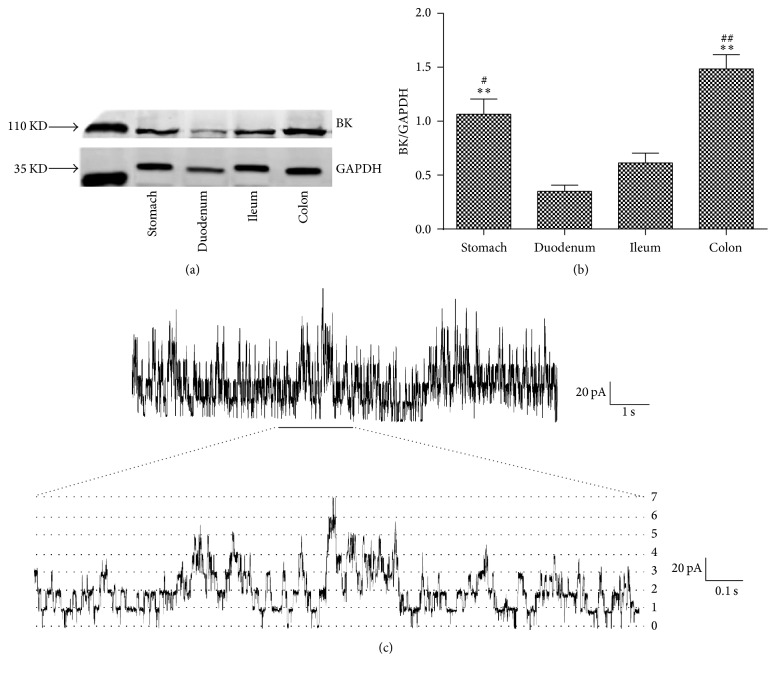
Expression of BK_Ca_ in smooth muscle tissues from different GI segments of rats. (a) and (b) Protein expression levels of BK_Ca_ in GI tract. All values are shown as means ± SEM, *n* = 4; ^*∗∗*^ *P* < 0.01 versus duodenum; ^#^ *P* < 0.05 versus ileum; ^##^ *P* < 0.01 versus ileum. (c) A single-channel recording was performed in high-K^+^ solutions in the cell-attached mode at +100 mV. The lower diagram is an extension of the underlined part of the upper diagram. Seven open levels were recorded in the patch.

To confirm that BK_Ca_ play an important role in colonic smooth muscle, the expression levels of BK_Ca_ in smooth muscle layers isolated from SD rat stomach, duodenum, ileum, and colon were detected first. Bar graphs show that BK_Ca_ expression was significantly higher in the colonic smooth muscle than other segments (Figures 1(a) and 1(b); *n* = 4,* P* < 0.05).

The single-channel activities of BK_Ca_ on colonic SMCs were identified in the symmetrical K^+^ solution then. The channels were activated by depolarization with a single-channel conductance of 210 ± 10 pS (*n* = 10) at +100 mV. The currents were abolished by 100 nM ChTX (data not shown). Multiple open levels could be recorded at +100 mV (Figure 1(c)). The lower diagram is an extension of the underlined part of the upper diagram. Seven open levels were recorded which means at least seven channels existed in the patch. There were 3–7 open levels in each patch (data not shown), which indicated the density of BK_Ca_ was high in colonic SMCs.

### 3.2. Mechanosensitivity of BK_Ca_ in Rat Colonic SMCs

**Figure 2:**
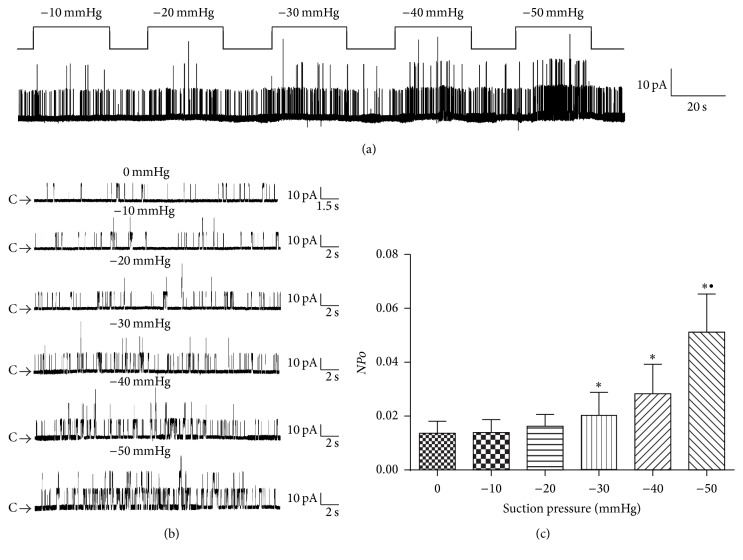
Activation of BK_Ca_ by membrane stretch in rat colonic SMCs. (a) The pressure-dependent activation of BK_Ca_ channels was recorded from a cell-attached patch. The patches were held at +60 mV. (b) The expanded data show the amplitude of the single-channel current was not changed compared with that in other panels (C: closed state). (c) Statistical data of BK_Ca_ channel open probability (*NPo*) challenged by negative pressure on cell-attached patch. Values are shown as means ± SEM, *n* = 5; ^*∗*^ *P* < 0.05 versus control group; ^∙^ *P* < 0.05 versus −30 mmHg group.

BK_Ca_ were continuously activated at +60 mV on cell-attached patches and membrane stretch was elicited by applying negative suction pressures ranging from −10 mmHg to −50 mmHg for 30 s with interval of 15 s. A representative patch is shown in Figure 2(a). The expanded data show the amplitude of the single-channel current was not changed while the channel activities increased as the increase of the negative suction pressures (Figure 2(b)). −10 mmHg and −20 mmHg had little effects on channel activities. *NPo* was increased obviously in response to −30 mmHg, −40 mmHg, and −50 mmHg negative suctions (Figure 2(c); *n* = 5,* P* < 0.05). These results suggest that BK_Ca_ in rat colonic SMCs could be activated by membrane stretch in a pressure-dependent manner.

### 3.3. Role of BK_Ca_ in the Myogenic Autoregulation of Colonic Smooth Muscle Strips Enduring Stretch

**Figure 3:**
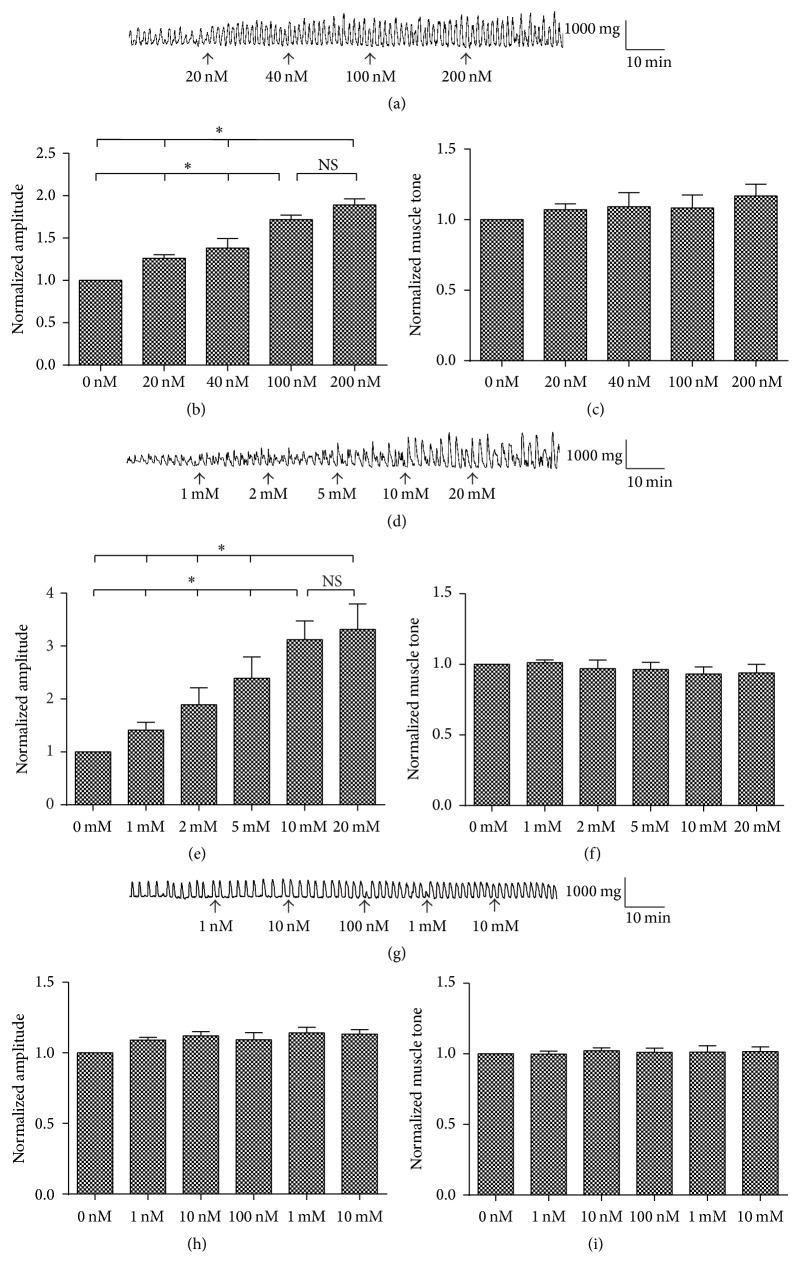
Effects of ChTX, TEA, and TTX on the muscle tone and contraction amplitude of colonic smooth muscle strips. (a) Effects of ChTX, a nonspecific blocker of BK_Ca_ channels, on the activities of colonic longitudinal muscle. (b) Active contraction amplitude increased dose-dependently by ChTX. (c) ChTX had no effect on the muscle tone of the colon. (d) The effects of TEA, a nonspecific potassium channel blocker, on the activities of colonic longitudinal muscle. (e) Active contraction amplitude increased by TEA. (f) TEA had no effect on the muscle tone of the colon. (g) The effect of TTX, a specific voltage-gated sodium channel blocker, on the activities of the colonic longitudinal muscle. (h) and (i) TTX had no effect on the active contraction amplitude and the muscle tone of the colon. Values are shown as means ± SEM, *n* = 6; ^*∗*^ *P* < 0.05.

We examined the dose-dependent effects of ChTX, TEA, and TTX on the muscle tone and contraction amplitude of colonic smooth muscle strips (Figure 3). Contraction amplitude increased after blocking BK_Ca_ by ChTX and the affect was dose-dependent (Figures 3(a) and 3(b); *n* = 6,* P* < 0.05). 100 nM ChTX had the same maximal affects as 200 nM, while blocking BK_Ca_ by ChTX had no effect on the muscle tone (Figures 3(a) and 3(c); *n* = 6,* P* > 0.05). Contraction amplitude increased after blocking potassium channels by TEA (Figures 3(d) and 3(e); *n* = 6,* P* < 0.05). Blocking potassium channels with TEA had no effect on the muscle tone (Figures 3(d) and 3(f); *n* = 6,* P* > 0.05). BK_Ca_ may be activated by both depolarization and increased cytoplasmic calcium during the active contraction process to contribute to repolarization and to regulate the contraction of colonic smooth muscle as a kind of negative feedback. Voltage-gated sodium channel blocker TTX was used to block the effect of enteric nervous system in this study. TTX had no effects on the contraction amplitude or on the muscle tone of colonic smooth muscle strips.

**Figure 4:**
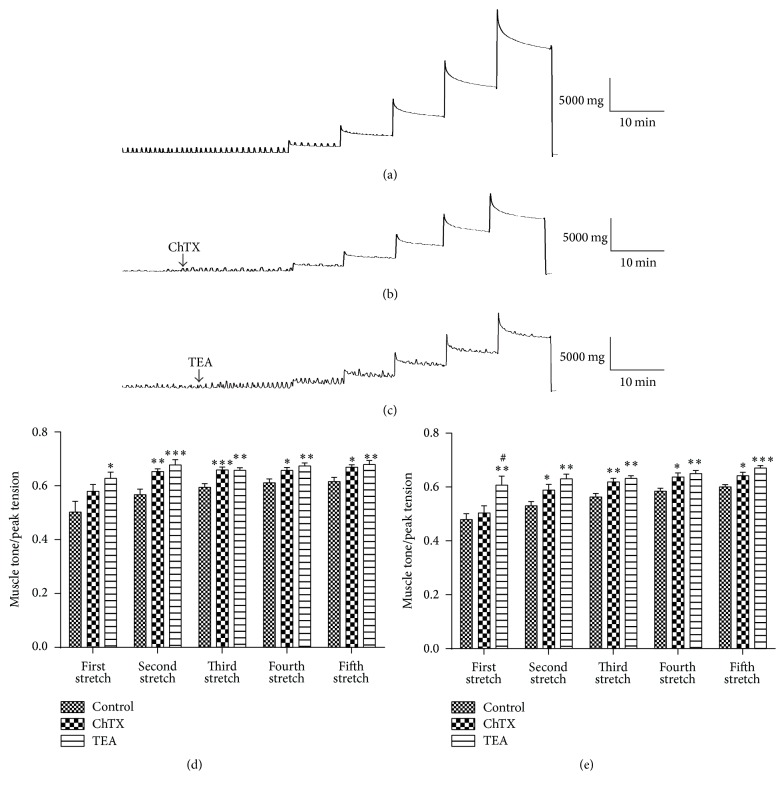
The tension changes of colon smooth muscle strips induced by stretch stimulation. (a) The longitudinal muscle tension changes in the control group after stretch stimulation. (b) The effect of ChTX on the tension changes induced by stretch. (c) The effect of TEA on the tension changes induced by stretch. (d) and (e) Statistical data of the ratio of the stable muscle tone and the relevant stretched peak tension for longitudinal muscle (d) and circular muscle (e), respectively. Values are shown as means ± SEM, *n* = 10; ^*∗*^ *P* < 0.05, ^*∗∗*^ *P* < 0.01, and ^*∗∗∗*^ *P* < 0.001 versus control group; ^#^ *P* < 0.05 versus ChTX group.

Effects of BK_Ca_ on the tension regulation induced by stretch were investigated. After equilibrating for 60 min, the colonic smooth muscle strips were exposed to ChTX (100 nM) or TEA (10 mM) for 15 min to block BK_Ca_. Then, the strips were stretched. Specifically the strip was elongated 1 mm for 10 min each time with a total 5 mm extension exerted on the strip. As shown in Figure 4(a), with the application of stretch stimulation, the muscle tone increased to reach a peak value following a gradual decrease to a relatively stable level. The ratio of stable muscle tone and stretched peak tension in the same step was calculated to reflect the capacity of colonic smooth muscle relaxation. A higher ratio reflected a decreased capability to relax. The ratio was significantly increased by ChTX and TEA in both longitudinal muscle strips (Figures 4(b), 4(c), and 4(d)) and circular muscle strips (Figure 4(e)) from the second-time stretch to the fifth-time stretch. These results indicated that BK_Ca_ were involved in the relaxation of GI tract induced by stretch.

**Figure 5:**
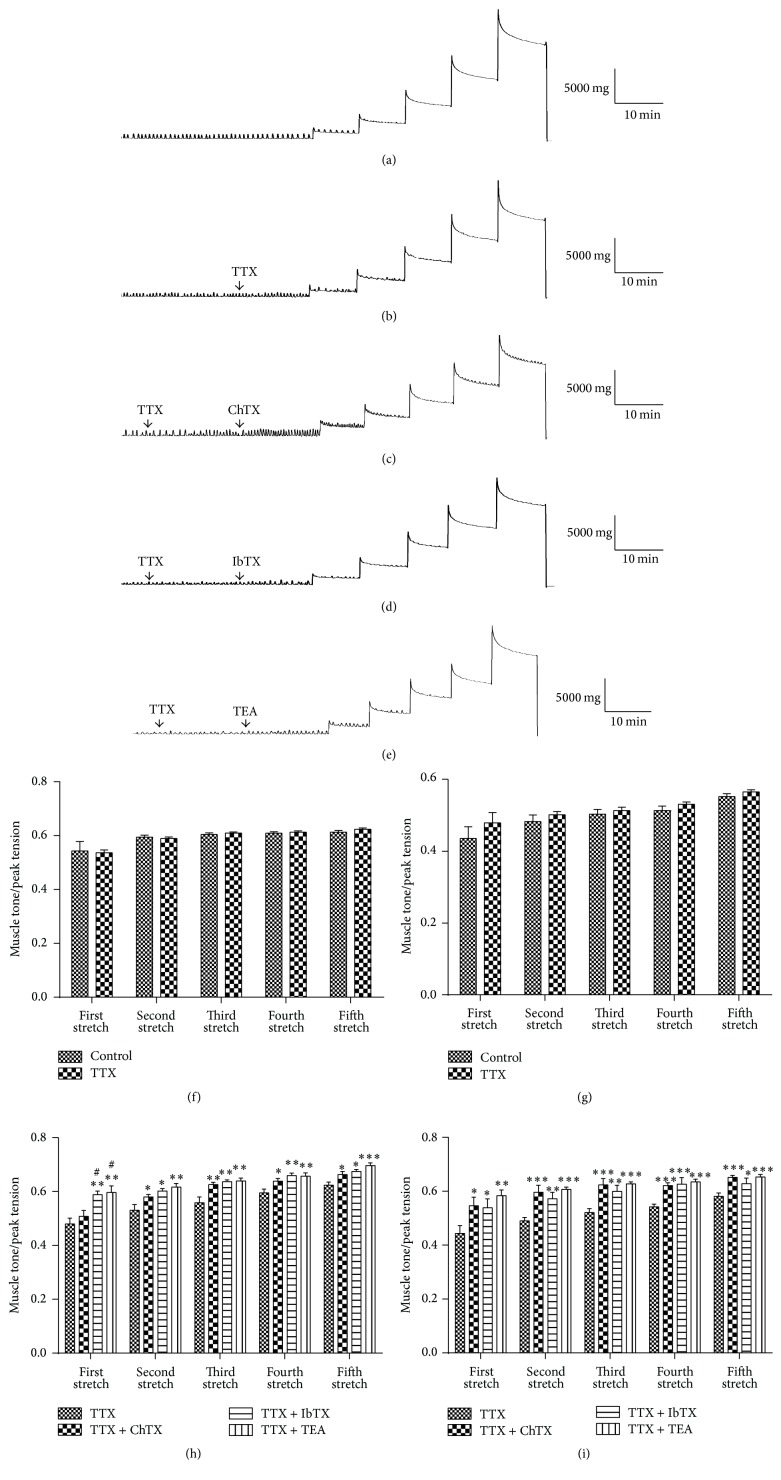
The tension changes induced by stretch stimulations after blocking the nervous system by TTX. (a) The longitudinal muscle tension changes induced by stretch stimulation in the control group. (b) The longitudinal muscle tension changes induced by stretch stimulation in the TTX group. (c) The effects of TTX + ChTX on the longitudinal muscle tension changes induced by stretch. (d) The effects of TTX + IbTX on the longitudinal muscle tension changes induced by stretch. (e) The effects of TTX + TEA on the stretch-induced longitudinal muscle tension changes. (f) and (g) Statistical data of the ratio of stable muscle tone and stretched peak tension from TTX and control groups for longitudinal muscle (f) and circular muscle (g). (h) and (i) Statistical data of the ratio of stable muscle tone and stretched peak tension from TTX, TTX + ChTX, TTX + IbTX, and TTX + TEA groups for longitudinal muscle (h) and circular muscle (i). Values are shown as means ± SEM, *n* = 9 for TTX, TTX + ChTX, and TTX + TEA groups and *n* = 6 for TTX + IbTX group; ^*∗*^ *P* < 0.05, ^*∗∗*^ *P* < 0.01, and ^*∗∗∗*^ *P* < 0.001 versus TTX group; ^#^ *P* < 0.05 versus TTX + ChTX group.

To exclude the influence of the enteric nervous system, TTX was used to block nerve activities and then the previous process was repeated (Figures 5(a) and 5(b)). TTX (100 nM) did not affect the ratio of muscle tone/peak tension in longitudinal or circular muscle strips enduring stretch (Figures 5(f) and 5(g)). The muscle strips were exposed to ChTX, IbTX, or TEA after blocking the nervous system by TTX and then stretched (Figures 5(c), 5(d), and 5(e)). The statistical results showed the ratio significantly increased in both longitudinal and circular muscle strips (Figures 5(h) and 5(i)) from the second-time stretch to the fifth-time stretch in ChTX, IbTX, and TEA groups. As a broad-spectrum blocking agent of Kv, TEA can block a variety of potassium channels including BK_Ca_ channels. ChTX, usually used as a nonspecific BK_Ca_ blocker, can also block other potassium channels such as intermediate conductance calcium-activated potassium channels (IK_Ca_) [19]. Because there are many other potassium channels besides BK_Ca_ in colonic SMCs [20–22], the BK_Ca_ specific blocker IbTX was used to confirm the role of BK_Ca_ and the results were similar to that of the TTX + ChTX group (Figure 5). These results indicated that activation of BK_Ca_ in colonic SMCs by stretch may be the underlying mechanism of the stretch-induced relaxation of the colonic smooth muscle strips.

## 4. Discussion

Many parts of the GI tract serve a reservoir function. The increase of GI contents does not cause a sustained rise in cavity pressure, which suggests relaxation may be induced by expanding GI wall, thus playing a role of storage [2]. Stretch-dependent increase in colon compliance might come from enteric nervous system that inhibits electrical excitability and contractile processes [10], while stretch stimulation applied on the GI wall may cause direct relaxation of GI smooth muscle without the involvement of enteric nervous system. However, the related mechanisms are unclear.

Studies have shown that SMCs of GI tract can respond to mechanical stimulation directly [4, 9, 23]. Mechanosensitive ion channels in the SMCs may play an important role in the process of sensing and transducing of mechanical stimuli. Mechanosensitive ion channels found in GI SMCs include swelling-activated chloride channels [24], stretch-activated nonselective cation channels [25], and calcium channels [26]. These channels are activated to generate inward currents and induce smooth muscle contraction. In the colon, stretch stimulation does not cause obvious contractile response and activation of outward currents may be necessary for stabilizing resting potential to remain in the relaxed state, yet little is known about the molecular basis [4, 27].

It has been known that BK_Ca_ exist in colonic SMCs. However, there are few studies comparing the expression of BK_Ca_ in different GI segment. We found that the expression levels of BK_Ca_ in colon, which has the storage function, are significantly higher than other GI segments, especially the small intestine, the main function of which is motion and absorption. Patch-clamp results also indicate the high density of BK_Ca_ in colonic SMCs. The abundant distribution suggests that BK_Ca_ may play an important role in colonic motility regulation.

Mechanosensitivity of BK_Ca_ has been reported [15–18]. In our previous studies, we have demonstrated that stretch is a BK_Ca_ gating factor independent of transmembrane potential and intracellular Ca^2+^ [18]. In this study, BK_Ca_ currents in rat colonic SMCs were recorded in cell-attached mode with 140 mM potassium in pipette solution and extracellular solution making the resting potential at around 0 mV. Suction pressure was applied to a small area of cell membrane contacted with the electrode. This is a conventional method for the study of mechanosensitivity of ion channels [28, 29]. We found BK_Ca_ in rat colonic SMCs could be activated by stretch intensity dependently. Although it is difficult to evaluate the physiological relevance of the pressures and holding potentials used, the mechanosensitivity of BK_Ca_ is conclusive. However, as calcium sensitive potassium channels, BK_Ca_ currents with intracellular calcium signals detection together will provide more valuable information. According to our previous [18] and current studies, BK_Ca_ are highly expressed in colonic SMCs both in rats and mice and are of very similar mechanosensitivity. There are no differences between species, suggesting a common and important role of BK_Ca_ in regulating colonic motility. Membrane hyperpolarization induced by mechanical activation of BK_Ca_ may well explain smooth muscle relaxation rather than contract in response to stretch.

It was reported that BK_Ca_ was involved in the mobility regulation of colon and there was an increase in Bethanechol-induced contraction amplitude in colon with impaired BK_Ca_ [30]. To confirm the role of BK_Ca_ in colon motility regulation, muscle tone and contraction amplitude of colonic smooth muscle strips were recorded. In the current study, blocking BK_Ca_ by ChTX enhanced active contraction but had no effects on muscle tone under basic condition. It is reasonable to speculate that BK_Ca_ were activated by both depolarization and increased cytoplasmic calcium as the action potential occurred. BK_Ca_ may play a negative feedback role to repolarize membrane potential and stop calcium inflow. While there were no BK_Ca_ activities at rest, ChTX had no effects on muscle tone.

Even though there are many researchers suggesting that BK_Ca_ was involved in the mobility regulation of colon, the role of BK_Ca_ in the regulation of muscle tension induced by stretch stimulation is seldom reported at organ level. Stretch-induced relaxation but not contraction of colonic smooth muscle is of significance for its storage role. Our previous study showed that stretch is an independent gating factor for BK_Ca_ activation [18]. However, it is unknown whether BK_Ca_ can be activated at tissue level by stretch independent on the occurrence of active contraction and plays any role in muscle tone regulation of colon enduring stretch. Tension response of colon to stretch and the role of BK_Ca_ in it were studied in the current study. The results showed that stretch induced relaxation of rat colonic strips. The relaxation capability of rat colonic muscle strips enduring stretch was impaired by blocking BK_Ca_ with ChTX. There was no significant difference between the ChTX and TEA groups which indicated BK_Ca_ was the main component of potassium channels involved in the process of tension regulation induced by stretch in colon. This result indicates that BK_Ca_ may be activated by stretch directly to stabilize resting potential and maintain the relaxed state.

It is reported that localized distension activates enteric reflex to evoke GI relaxation [8, 11, 12]. However, our previous study provides evidence that BK_Ca_ in SMCs can be activated by stretch directly without the involvement of enteric nervous system [18]. The responses of colonic smooth muscle strips to stretch were studied* in vitro*, excluding effects of external venous and humoral factors. TTX was used to block enteric nervous activities. Results showed the relaxation response of colonic strips to stretch was not changed by TTX. Blocking of BK_Ca_ using ChTX or IbTX still increased the muscle tone/peak tension ratio of colonic smooth muscle strips being stretched, suggesting the nervous system did not appear to be necessary and the activation of BK_Ca_ had a role in this process.

In conclusion, we report, for the first time, that BK_Ca_ are abundantly expressed in the colon more than other GI tracts, and there is stretch-induced relaxation in colon and mechanosensitive BK_Ca_ function in it. The sensation and transduction of mechanical signals have important impacts on GI motility functions. Our study highlights a novel mechanism of GI motility controlling. The disturbed mechanical environment may be key pathogenic factors in GI motility disorders involving organ distention and the mechanosensitivity of BK_Ca_ is worthy of more attention.
